# 

**Published:** 1883-07-20

**Authors:** 


### TABLE OF CONTENTS.

ORIGINAL AND SELECTED ARTICLES.

American Medical Association, 241. The Opium Habit, by W. D. Wilhite, M. D., 246. On the Importance of Recognizing the Conditions known as Sthenic and Asthenic, etc., by Harvey L Byrd, M.D., Baltimore, 249. A Trustworthy Combination of Coca, Viburnum and Celery, etc., by Wm. B. Hazard, M. D., 253. Swallowing a Silver Half-dollar—Death, 256. Fatal Effects from the Bee Sting. Cure for Squint without Operation, 257.

ABSTRACTS AND GLEANINGS.

Bromide of Sodium & Bromide of Potassium, 258. Boroglyceride in the Treatment of Diseases of the Eye and Ear, 259. Hemorrhagic Diathesis, 260. Viability of the Premature Fetus, 261. Percussion of the Colon in Diarrhœa, 262. Iodoform in Spray Form; The Teeth in Diabetes, 263. Diphtheria; Local Anæsthesia, 264. Six Years' Experience in the Treatment of Syphilis; Vaccination with the Saliva of a Calf, 265. Artificial Human Milk; Mullein in Phthisis, 266. Carbolic Acid in Piles and Fistulæ; A Hydrophobia Cure: Unpleasant Effects of Quinine; Hydrastin in Laryngeal Phthisis, 267. Removal of Warts by Cauterization; The Treatment of Croup; Swallowing of Shot and Insufflation in the Treatment of Ileus; Glycerine; Lancing the Gums, 268. How Long Should the Subjects of Contagious Diseases be Isolated? Death from Petroleum by Suffocation; Quinine Intoxication, 269. Baer's Tonic; Turpentine in Diphtheria; Sub-mucous Chloroform Injections in Toothache; Medical Treatment of Obstinate Neuralgia; A Smart Doctor, 270.

SCIENTIFIC ITEMS.

How to Act in a Tornado, 271. Photography and Pock-marks; Cheap Water Filter; Sun-Spot Laws: A Mastodon Graveyard, 272.

PRACTICAL NOTES AND FORMULÆ.

Cathartic for Infants; Aloes Externally, 273. Tannate of Cannabine; Grindelia Robusta in Asthma; Eczema; Clergyman's Sore Throat, 274. Kalsomining Fluid; A New Invisible Ink; Treatment of Consumptives by Causing them to Breathe an Antiseptic Atmosphere; A Palatable Cough Mixture; Bleeding Piles, 275.

EDITORIALS AND MISCELLANEOUS.

Editorial Notices; American Surgical Association; Tri-State Medical Association; American Medical Editors, 276. Journalism as a Teacher, 277. Medical Codes, etc., 278. The American Medical Association—The Code: Cholera; Pamphlets received, 279. Book Notices; Special Notices, 280.

